# Regional lymph node metastasis as a risk factor for recurrence in Borrmann type II gastric cancer: particular significance of the no. 7 lymph node

**DOI:** 10.3389/fonc.2026.1797970

**Published:** 2026-05-08

**Authors:** Hongfa Wei, Weidong Chen, Feiran Zhang, Yifeng Zheng, Changhua Zhang

**Affiliations:** 1 Department of General Surgery, The First Affiliated Hospital of Shantou University Medical College, Shantou, China; 2 Department of Gastrointestinal Surgery, The Seventh Affiliated Hospital, Sun Yat-sen University, Shenzhen, China

**Keywords:** Borrmann type II, clinicopathological characteristics, gastric cancer, lymph node metastasis, recurrence

## Abstract

**Background:**

The Borrmann classification is a well-established system for categorizing gastric cancer (GC). Although Borrmann type II GC is generally associated with favorable outcomes, a subset of patients still experiences recurrence with a poor prognosis. This study identified clinicopathological factors in Borrmann type II GC patients who are surgically targetable, with the aim of guiding gastrointestinal surgeons and improving patient prognosis.

**Methods:**

A total of 1,824 patients with GC who underwent radical gastrectomy at the First Affiliated Hospital of Sun Yat-sen University (2007-2019) were retrospectively analyzed, with an additional 197 patients from the First Affiliated Hospital of Shantou University Medical College (2014-2017) used for validation.

**Results:**

In the entire cohort, 440 patients (24.1%) were identified as having type II GC. Compared with other types of GC, type II GC is associated with more favorable overall survival. This subtype is characterized by a smaller tumor size and a lower incidence of serosal invasion and lymph node metastasis. Compared with nonrecurrent cases, recurrent cases more commonly involved tumors located in the upper stomach, demonstrated increased lymphatic spread, and were associated with more advanced pTNM staging. Notably, among the second-tier lymph node stations, tumor involvement of the No. 7 lymph nodes emerged as an important risk factor for recurrence. The peritoneum was the most frequent site of recurrence.

**Conclusions:**

Although type II GC patients usually have a favorable prognosis, disease recurrence dramatically impairs survival. Notably, the involvement of lymph node station No. 7 was an independent predictor of recurrence, indicating the need for meticulous lymphadenectomy and intensive postoperative surveillance.

## 1 Introduction

GC is the fifth most common malignancy worldwide, with over 1 million new cases recorded annually. With more than 700,000 global deaths in 2018, GC represents the third most lethal cancer, highlighting its critical mortality burden (1). The prognosis of patients with GC undergoing radical resection is primarily determined by clinicopathological tumor characteristics (2–4). Among these factors, tumor recurrence remains one of the most critical threats to patient outcomes (5).

The Borrmann classification, which can be conveniently assessed via endoscopy prior to surgery, is widely used by gastrointestinal surgeons and pathologists to predict the prognosis of GC patients (6, 7). Most studies have focused on Borrmann type IV, as it represents the subtype with the poorest prognosis (8–10). In contrast, limited research has been conducted on the clinicopathological features of Borrmann type II GC, especially in patients who experience tumor recurrence. Borrmann type II is characterized by ulcerated lesions with well-defined, elevated margins and is generally associated with a relatively favorable prognosis (11–13). However, a subset of these patients still suffer poor outcomes due to recurrence.

This research analyzed 1,824 patients with GC treated at the First Affiliated Hospital of Sun Yat-sen University (2007–2019) and validated the findings in 197 patients from the First Affiliated Hospital of Shantou University Medical College (2014–2017). In our study, Borrmann type II GC patients had favorable survival but experienced poor outcomes due to recurrence. Because Borrmann type II GC is generally associated with a favorable outlook, there is a clinical risk of underestimating the threat of recurrence in this specific population. Therefore, this exploratory study aimed to identify the clinicopathological factors that contribute to these unexpected adverse outcomes in Borrmann type II gastric cancer patients, with a particular emphasis on the impact of lymph node involvement. By highlighting these hidden risk factors, we seek to provide gastrointestinal surgeons with insights to optimize the extent of lymphadenectomy and establish more intensive postoperative surveillance for high-risk type II patients.

## 2 Materials and methods

### 2.1 Data sources

The medical records of the GC patients were retrospectively reviewed. The inclusion criteria were as follows: (1) primary GC treated with surgical resection; (2) availability of detailed pathological reports and survival information; and (3) absence of chronic or severe diseases in major organs (e.g., kidney, liver, heart). The exclusion criteria included (1) a diagnosis of gastroesophageal junction tumors, gastric carcinoid tumors, gastric lymphoma, or gastrointestinal stromal tumors; (2) patients who received preoperative neoadjuvant chemotherapy or radiotherapy; (3) incomplete clinical or pathological data; (4) missing survival information; and (5) a history of other malignancies.

A total of 1,824 patients from the First Affiliated Hospital of Sun Yat-sen University (2007–2019) constituted the primary test cohort, whereas 197 patients from the First Affiliated Hospital of Shantou University Medical College (2014–2017) served as the validation cohort. According to the Borrmann classification, the Sun Yat-sen cohort comprised type I (n = 61), type II (n = 440), type III (n = 1,090), and type IV (n = 233) patients, whereas the Shantou cohort comprised type I (n = 9), type II (n = 44), type III (n = 123), and type IV (n = 20) patients (11). All patients included in this study underwent radical gastrectomy with standard D2 lymphadenectomy. The surgical procedures and the extent of lymph node dissection, including the clearance of the No. 7 lymph node station along the left gastric artery, were completely standardized across both centers in strict accordance with the Japanese Gastric Cancer Association (JGCA) guidelines (14).

### 2.2 Diagnostic criteria

Anatomically, the stomach is subdivided into three major regions: the cardia, corpus, and antrum. The terms “upper stomach” and “lower stomach” are used when the tumor invades the gastric cardia or antrum, respectively. All patients underwent thoracic and abdominal CT scans with both oral and intravenous contrast, along with a barium meal combined with gastroscopy of the upper gastrointestinal tract to assess tumor size. *In vivo* diagnosis of GC was conducted via endoscopic optical biopsy. The macroscopic classification of GC was performed according to the Borrmann classification, which categorizes tumors into four types on the basis of gross morphology: type I (polypoid, nonulcerated) lesions present a broad base without ulceration. Type II (Ulcerated, Elevated) lesions are characterized by sharply demarcated margins and raised edges. Type III (ulcerated, infiltrative) are defined as an ulceration with diffuse infiltration at the base. Type IV (diffuse infiltration) are identified by diffuse infiltration of the gastric wall without a distinct ulcer or mass. Histopathological grading followed WHO criteria, categorizing tumors as well, moderately, or poorly differentiated (15). Tumor staging was based on the American Joint Committee on Cancer (AJCC)8th edition criteria (16). The regional lymph nodes were categorized according to the JGCA guidelines as follows: No. 1 (right paracardial), No. 2 (left paracardial), No. 3 (lesser curvature), No. 4 (left greater curvature), No. 5 (suprapyloric), No. 6 (infrapyloric), No. 7 (along the left gastric artery), No. 8 (along the common hepatic artery), No. 9 (around the celiac axis), No. 10 (splenic hilum), No. 11 (along the splenic artery), and No. 12 (within the hepatoduodenal ligament). Furthermore, extra-regional lymph nodes, whose involvement is classified as distant metastasis (M1) rather than regional disease, were also evaluated, including No. 13 (posterior to the pancreas), No. 14 (adjacent to the superior mesenteric vessels), No. 15 (around the middle colic vessels), and No. 16 (para-aortic region) (14).

### 2.3 Statistical analysis

The primary endpoint of this study was overall survival (OS), calculated from the date of surgery to the date of death from any cause or the last follow-up. The secondary endpoint was postoperative recurrence, defined as the first evidence of tumor relapse identified via radiological imaging (CT/MRI), endoscopy, or histopathological confirmation during the surveillance period.

Medians and interquartile ranges were used for continuous variables, whereas categorical variables were reported as frequencies and proportions. Survival curves were estimated via the Kaplan–Meier method, and differences between groups were evaluated via the log-rank test. The chi-square test was applied to analyze categorical variables, whereas the nonparametric Mann–Whitney U test was used for continuous variables. For univariate analysis of clinicopathological factors, chi-square tests were employed. Independent prognostic factors were identified through multivariate Cox regression analysis. Kendall’s tau-b correlation coefficient was calculated to assess associations among ranked variables. Hazard ratios (HRs) and corresponding 95% confidence intervals (CIs) for time-to-event outcomes were estimated via Cox proportional hazards regression. A two-sided P value < 0.05 was considered statistically significant. Additionally, to verify the stability of the results, we applied a bootstrap resampling procedure with 5000 iterations in the Firth logistic regression analysis. This approach allowed us to assess the robustness of the statistical estimates and their variability across different samples. All the statistical analyzes were performed via SPSS software version 22.0 (SPSS Inc., Chicago, IL, USA). Graphs were generated via GraphPad Prism version 8.0.2 (GraphPad Software, San Diego, CA, USA).

## 3 Results

### 3.1 Lower proportion of risk factors in type II GC

**Figure 1:**
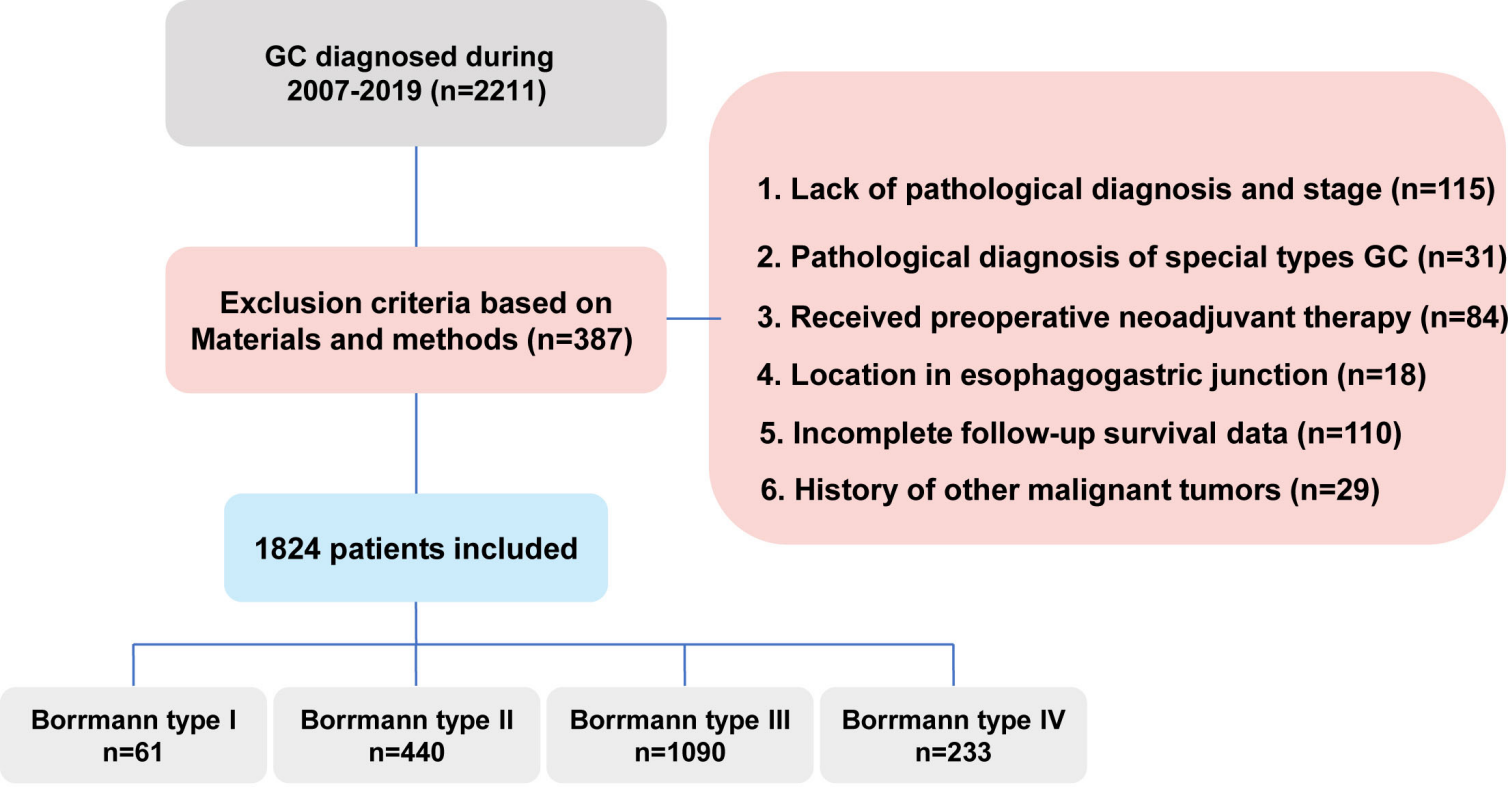
Flowchart of the selection of GC patients in the database.

**Figure 2:**
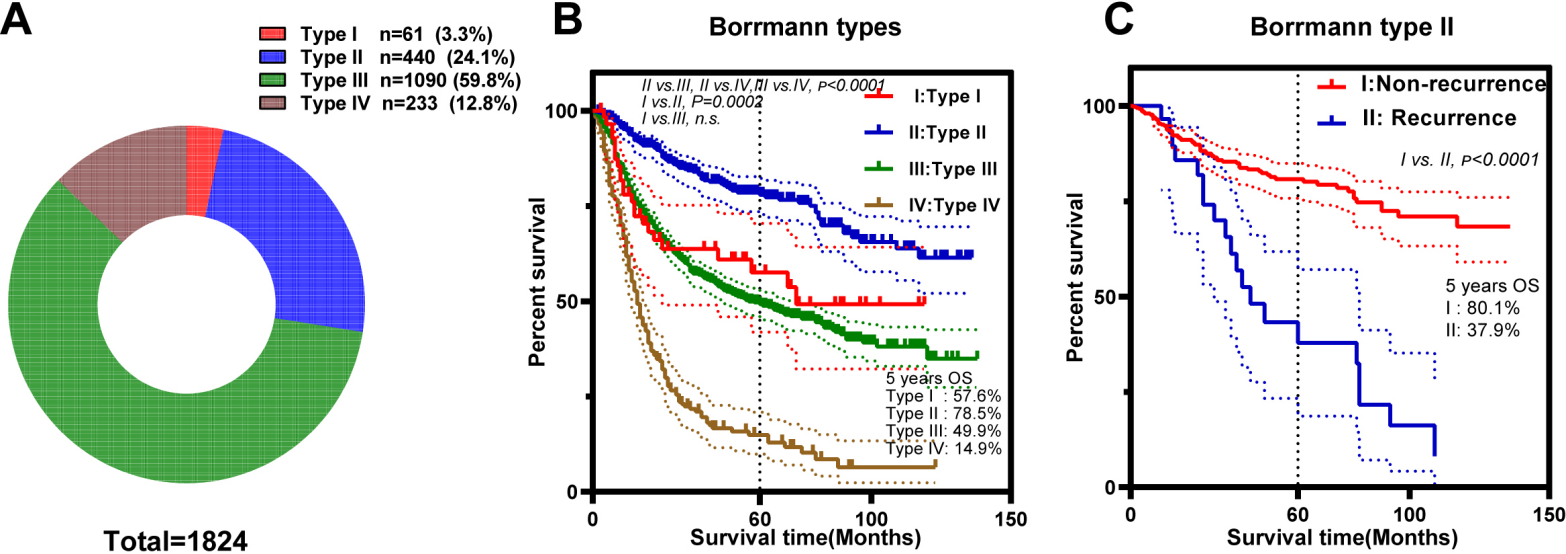
The general characteristics of the Borrmann types. **(A)** Constitution of different Borrmann types. **(B)** Kaplan–Meier survival curves according to Borrmann type. **(C)** Kaplan–Meier survival curves of the patients in the Borrmann type II recurrent and nonrecurrent groups.

As illustrated in the flowchart (**Figure 1**), a total of 1,824 GC patients were included on the basis of predefined exclusion criteria. Among them, 61 patients (3.3%) were classified as type I, 440 (24.1%) as type II, 1,090 (59.8%) as type III, and 233 (12.8%) as type IV (**Figure 2A**; **Table 1**).

**Table 1:** Clinicopathological characteristics and survival outcomes of gastric cancer patients from the First Affiliated Hospital of Sun Yat-sen University (2007--2019) according to the Borrmann classification.

2007-2019	Borrmann type	I		II		III		IV		p value
Proportion	1824	61	(3.3%)	440	(24.1%)	1090	(59.8%)	233	(12.8%)	
Survival or death rate(n)										
Live	1079	38	(63.3%)	351	(79.8%)	634	(58.2%)	56	(24.0%)	0.0021#
death	745	23	(37.7%)	89	(20.2%)	456	(41.8%)	177	(76.0%)	<0.0001*
Age, years	59(50-66)	62(54-70)		62(54-70)		60(52-67)		57(47-65)		
<45	259	4	(6.6%)	70	(15.9%)	139	(12.8%)	46	(19.8%)	0.0416#
45-59	671	19	(31.1%)	164	(37.3%)	398	(36.5%)	90	(38.6%)	n.s.*
≥60	894	38	(62.3%)	206	(46.8%)	553	(50.7%)	97	(41.6%)	
Gender										
Male, n (%)	1187	49	(80.3%)	273	(62.0%)	737	(67.6%)	128	(54.9%)	0.0052#
Female, n (%)	637	12	(19.7%)	167	(38.0%)	353	(32.4%)	105	(45.1%)	0.0374*
Hospitalization, days	10(8-13)	10(8-14)		10(8-13)		10(8-13)		10(8-14)		
≤8, n (%)	597	19	(31.2%)	169	(38.4%)	340	(31.2%)	69	(29.6%)	n.s.#
9-15, n (%)	942	31	(50.8%)	221	(50.2%)	573	(52.6%)	117	(50.2%)	0.0059*
>15, n (%)	285	11	(18.0%)	50	(11.4%)	177	(16.2%)	47	(20.2%)	
Peritoneal metastasis, n (%)										
P0	1616	55	(90.2%)	431	(98.0%)	972	(89.2%)	158	(67.8%)	0.0008#
P1	208	6	(9.8%)	9	(2.0%)	118	(10.8%)	75	(32.2%)	<0.0001*
Hepatic metastasis, n (%)										
H0	1787	60	(98.4%)	436	(99.1%)	1064	(97.6%)	227	(97.4%)	n.s.#
H1	37	1	(1.6%)	4	(0.9%)	26	(2.4%)	6	(2.6%)	n.s.*
Tumor location, n (%)										
Upper stomach	454	26	(44.8%)	93	(21.5%)	286	(27.1%)	49	(30.4%)	0.0005#
Corpus	308	8	(13.8%)	85	(19.7%)	202	(19.1%)	13	(8.1%)	n.s.*
Lower stomach	944	24	(41.4%)	254	(58.8%)	567	(53.8%)	99	(61.5%)	
Tumor Size (cm)	4.0(2.5-5.0)	4.00(3.0-6.00)		4.00(3.00-6.00)		4.00(3.00-5.00)		6.00(5.00-8.00)		
Tumor Size, n (%)										
<3	433	10	(19.2%)	229	(60.3%)	181	(18.6%)	13	(7.0%)	<0.0001#
3-6	839	25	(48.1%)	140	(36.8%)	618	(63.6%)	56	(30.3%)	<0.0001*
≥6	317	17	(32.7%)	11	(2.9%)	173	(17.8%)	116	(62.7%)	
Histological type										
well-differentiation	27	3	(6.1%)	16	(4.8%)	7	(0.8%)	1	(0.5%)	n.s.#
moderately differentiation	342	16	(32.7%)	102	(30.6%)	205	(23.1%)	19	(9.5%)	<0.0001*
poor-differentiation	1101	30	(61.2%)	215	(64.6%)	677	(76.1%)	179	(90.0%)	
Pathological Subtypes										
Adenocarcinoma	1123	24	(70.6%)	264	(94.0%)	708	(90.5%)	127	(87.0%)	<0.0001#
Others	120	10	(29.4%)	17	(6.0%)	74	(9.5%)	19	(13.0%)	n.s.*
T stage										
T2	437	15	(27.8%)	232	(56.6%)	178	(17.1%)	12	(5.7%)	0.0027#
T3	973	31	(57.4%)	159	(38.8%)	670	(64.8%)	113	(53.3%)	<0.0001*
T4	302	8	(14.8%)	19	(4.6%)	188	(18.1%)	87	(41.0%)	
pTNM stage										
I	228	9	(14.8%)	146	(33.9%)	67	(6.2%)	6	(2.6%)	<0.0001#
II	340	17	(27.8%)	107	(24.8%)	194	(18.0%)	22	(9.5%)	<0.0001*
III	897	25	(41.0%)	165	(38.3%)	613	(56.7%)	94	(40.7%)	
IV	338	10	(16.4%)	13	(3.0%)	206	(19.1%)	109	(47.2%)	
Lymphatic metastasis	2(0-9)	2(0-5)		2(0-5)		3(0-9)		8(1-19)		
N0	634	27	(45.0%)	259	(58.9%)	292	(26.8%)	56	(24.1%)	0.0418#
Node-positive	275	33	(55.0%)	181	(41.1%)	797	(73.2%)	176	(75.9%)	<0.0001*
Laboratory findings										
Hematologic										
Leukocyte count, 10^9^/l	5.89(4.87-7.30)	6.16(5.16-7.16)		6.16(5.16-7.16)		5.94(4.91-7.45)		5.72(4.76-6.96)		
<4 × 10^9^/l, n (%)	162	4	(7.0%)	38	(9.3%)	92	(8.8%)	28	(12.8%)	n.s.#
4–10 × 10^9^/l, n (%)	1486	49	(86.0%)	352	(86.5%)	903	(86.1%)	182	(83.5%)	n.s.*
>10 × 10^9^/l, n (%)	82	4	(7.0%)	17	(4.2%)	53	(5.1%)	8	(3.7%)	
Hemoglobin, g/l	99.75(122-137)	115(81-137)		115(81-137)		121(99-136)		118(99-132)		
>120	919	28	(49.1%)	252	(61.9%)	535	(51.1%)	104	(47.7%)	0.0045#
90-120	528	11	(19.3%)	101	(24.8%)	341	(32.5%)	75	(34.4%)	0.0027*
<90	283	18	(31.6%)	54	(13.3%)	172	(16.4%)	39	(17.9%)	
Platelet count, 10^9^/l	240(191-301)	232(186-344)		232(187-344)		243.00(193-307.00)		242(194-308)		
<100 × 109/l, n (%)	47	1	(1.8%)	7	(1.7%)	29	(2.8%)	10	(4.6%)	0.0112#
100–300 × 109/l, n (%)	1246	37	(64.9%)	330	(81.5%)	731	(70.0%)	148	(67.9%)	<0.0001*
>300 × 109/l, n (%)	432	19	(33.3%)	68	(16.8%)	285	(27.2%)	60	(27.5%)	
Albumin, g/l	39.00(35.88-42.00)	38.00(35.70-40.60)		38.00(35.70-40.60)		38.8(35.60-41.80)		38.00(35.00-41.00)		
<40	1018	42	(73.7%)	209	(51.4%)	626	(59.7%)	141	(64.7%)	0.0015#
≥40	712	15	(26.3%)	198	(48.6%)	422	(40.3%)	77	(35.3%)	0.0037*
Globulin, g/l	25.95(23.00-29.00)	25.00(22.30-28.60)		25.00(22.30-28.60)		26.00(23.00-29.00)		25.80(22.73-28.18)		
≤20	185	8	(14.0%)	41	(10.1%)	114	(10.9%)	22	(10.1%)	n.s.#
>20	1545	49	(86.0%)	366	(89.9%)	934	(89.1%)	196	(89.9%)	n.s.*
Bilirubin, umol/l	10.20(7.70-13.70)	10.30(7.68-15.38)		10.30(7.68-15.38)		9.90(7.28-13.10)		10.10(7.55-14.25)		
≤17.1	1041	27	(84.4%)	246	(84.2%)	651	(89.5%)	117	(81.8%)	n.s.#
>17.1	153	5	(15.6%)	46	(15.8%)	76	(10.5%)	26	(18.2%)	0.0185*
ALT, IU/l	15.00(10.50-23.00)	15.50(10.50-24.75)		15.50(10.50-24.75)		14.00(10.00-23.00)		13.00(10.00-23.00)		
≤40	1119	30	(93.7%)	275	(94.2%)	684	(94.1%)	130	(90.9%)	n.s.#
>40	75	2	(6.3%)	17	(5.8%)	43	(5.9%)	13	(9.1%)	n.s.*
Creatine, umol/l	76.00(62.00-87.00)	80.00(64.25-94.00)		80.00(64.25-94.00)		76.00(62.00-87.00)		70.00(58.50-83.00)		
<133	1178	31	(96.9%)	288	(98.6%)	719	(98.9%)	140	(97.9%)	n.s.#
≥133	16	1	(3.1%)	4	(1.4%)	8	(1.1%)	3	(2.1%)	n.s.*
Glucose, mmol/l	4.80(4.40-5.40)	4.90(4.40-5.60)		4.90(4.40-5.60)		4.80(4.40-5.40)		4.70(4.30-5.20)		
≤6.1	1515	53	(93.0%)	364	(89.4%)	907	(86.5%)	191	(87.6%)	n.s.#
>6.1	215	4	(7.0%)	43	(10.6%)	141	(13.5%)	27	(12.4%)	n.s.*
AFP, ug/l	2.80(2.01-3.91)	2.92(2.03-4.01)		2.92(2.03-4.01)		2.88(2.02-4.12)		2.63(1.94-3.63)		
≤25	1690	57	(100.0%)	403	(99.0%)	1015	(96.9%)	215	(98.6%)	n.s.#
>25	40	0	(0.0%)	4	(1.0%)	33	(3.1%)	3	(1.4%)	0.0185*
CEA, ug/l	2.19(1.33-4.09)	2.24(1.48-4.92)		2.24(1.48-4.92)		2.31(1.35-4.62)		2.18(1.27-4.83)		
≤25	1390	44	(77.2%)	356	(87.5%)	824	(78.6%)	166	(76.1%)	0.0001#
>25	340	13	(22.8%)	51	(12.5%)	224	(21.4%)	52	(23.9%)	0.0351*
CA125, U/ml	10.40(7.30-16.00)	10.25(6.85-17.93)		10.25(6.85-17.93)		10.40(7.30-16.00)		14.10(8.40-22.88)		
≤35	1621	50	(87.7%)	393	(96.6%)	988	(94.3%)	190	(87.2%)	0.0026#
>35	109	7	(12.3%)	14	(3.4%)	60	(5.7%)	28	(12.8%)	n.s.*
CA19-9, U/ml	8.18(3.24-22.66)	11.52(3.32-73.61)		11.52(3.32-73.61)		8.48(3.23-25.74)		9.65(3.68-40.58)		
<37	1426	42	(73.7%)	373	(91.6%)	847	(80.8%)	164	(75.2%)	<0.0001#
≥37	304	15	(26.3%)	34	(8.4%)	201	(19.2%)	54	(24.8%)	<0.0001*
SCC, ug/l	0.70(0.50-0.90)	0.70(0.60-1.23)		0.70(0.60-1.23)		0.70(0.50-1.00)		0.60(0.40-0.90)		
≤1.5	1637	47	(82.5%)	390	(95.8%)	988	(94.3%)	212	(97.2%)	<0.0001#
>1.5	93	10	(17.5%)	17	(4.2%)	60	(5.7%)	6	(2.8%)	n.s.*
BMI	0.21(0.19-0.24)	0.21(0.19-0.24)		0.21(0.19-0.24)		0.21(0.19-0.24)		0.20(0.18-0.22)		
<0.185 (low weight)	253	10	(23.3%)	41	(12.3%)	155	(17.1%)	47	(26.6%)	n.s.#
0.185-0.25 (normal)	1015	27	(62.7%)	241	(72.6%)	629	(69.4%)	118	(66.6%)	n.s.*
0.25-0.3 (overweight) & >0.3 (obesity)	190	6	(14.0%)	50	(15.1%)	122	(13.5%)	12	(6.8%)	

#Chi-square tests were used to compare Borrmann type II and Borrmann type I with different indices.

*: Chi-square tests were used to compare Borrmann type II and Borrmann type III with different indices.

n.s: not significant.

Compared with patients with type I and type III disease, those with type II disease were significantly less common in several clinical subgroups. Specifically, the proportion of male patients was lower (62.0% vs. 80.3%, p = 0.0052; 62.0% vs. 67.6%, p = 0.0374), as was the incidence of peritoneal metastasis (2.0% vs. 9.8%, p = 0.0008; 2.0% vs. 10.8%, p < 0.0001). Additionally, patients with type II tumors had a lower frequency of large tumors (≥6 cm: 2.9% vs. 32.7%, p < 0.0001; 2.9% vs. 17.8%, p < 0.0001), T4 lesions (4.6% vs. 14.8%, p = 0.0027; 4.6% vs. 18.1%, p < 0.0001), lymphatic metastasis (41.1% vs. 55.0%, p = 0.0418; 41.1% vs. 73.2%, p < 0.0001), and advanced pTNM stages (3.0% vs. 16.4%, p < 0.0001; 3.0% vs. 19.1%, p < 0.0001).

Furthermore, elevated levels of carcinoembryonic antigen (CEA: 12.5% vs. 22.8%, p = 0.0001; 12.5% vs. 21.4%, p = 0.0351) and carbohydrate antigen 19-9 (CA19-9: 8.4% vs. 26.3%, p < 0.0001; 8.4% vs. 19.2%, p < 0.0001) were less frequently observed in type II patients (**Table 1**).

The validation cohort of 197 patients from Shantou University Medical College confirmed the key findings from the primary cohort, particularly with respect to tumor size, lymph node metastasis, and disease stage, further validating the clinicopathological characteristics of Borrmann type II gastric cancer. (**Supplementary Table 1**).

### 3.2 Clinicopathological characteristics do not confer protective effects in type II GC patients

**Figure 3:**
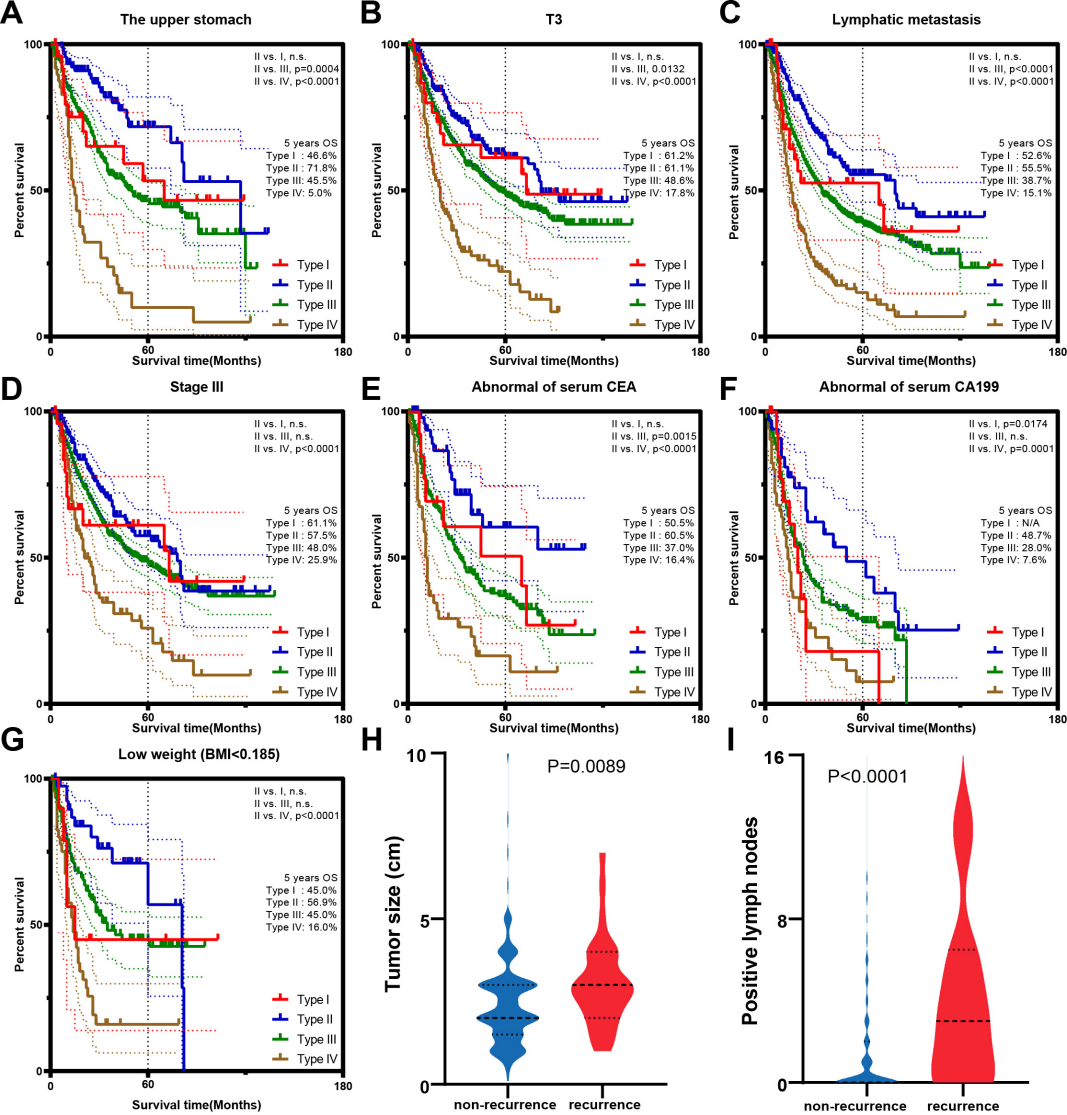
K–M survival curves of type II patients according to different prognostic factors. Curves according to **(A)** upper stomach, **(B)** T3, **(C)** lymphatic metastasis, **(D)** stage III, **(E)** CEA, **(F)** CA19-9, and **(G)** low BMI. Comparison between the recurrent and nonrecurrent groups. **(H)** Tumor size. **(I)** Positive lymph nodes.

Type I GC, characterized by its distinctive growth pattern toward the lumen rather than invasion of the deeper gastric wall, has traditionally been associated with a favorable prognosis compared with type II GC (17). However, in our cohort, type II patients had the highest overall survival (OS) among all Borrmann types, with a 5-year OS rate of 78.5% (vs. 57.6% for type I patients, p = 0.0002; **Figure 2B**), a pattern consistently observed in the validation cohort, where the 5-year OS rate was 79.5% for type II patients (vs. 44.4% for type I patients, p = 0.023; **Supplementary Figure 2A**). However, type II patients who experienced recurrence had markedly worse outcomes, with a 5-year OS of only 37.9%, compared with 80.1% in nonrecurrent patients (p < 0.0001; **Figure 2C**). Furthermore, certain clinicopathological factors failed to provide a prognostic advantage for type II patients compared with type I patients. First, among patients with tumors invading the upper third of the stomach, those with type II lesions exhibited better short- to midterm survival (≤5 years) postoperatively (71.8% vs. 46.6%, p < 0.05). However, this survival benefit was not sustained at the 10-year follow-up (p > 0.05; **Figure 3A**). Second, no survival advantage for type II patients was observed in the T3 subgroup, those with lymph node metastasis (node-positive), or those with stage III disease (**Figures 3B–D**). Third, elevated serum CEA levels were not associated with improved outcomes in type II patients (**Figure 3E**). In addition, patients with elevated CA19–9 levels had comparable prognoses between the type II subgroup and type III subgroup (**Figure 3F**). Certain results with limited clinical significance or small sample sizes were omitted from the detailed depiction and are presented in **Supplementary Figures 1A–P**.

Emerging evidence has highlighted the prognostic relevance of body mass index (BMI) in GC patients (18, 19). In our study, type II patients with low BMIs (<18.5 kg/m²) had significantly poorer outcomes than their normal-weight counterparts did (p = 0.0049; **Supplementary Figure 1Q**). Moreover, within the low BMI subgroup, type II patients did not have a survival advantage over those with type I or type III tumors (**Figure 3G**). Similarly, type II patients with hypoalbuminemia had worse prognoses than did those with normal serum albumin levels (p = 0.0042; **Supplementary Figure 1R**).

### 3.3 Risk factors and correlation between clinicopathological features of type II GC

**Figure 4:**
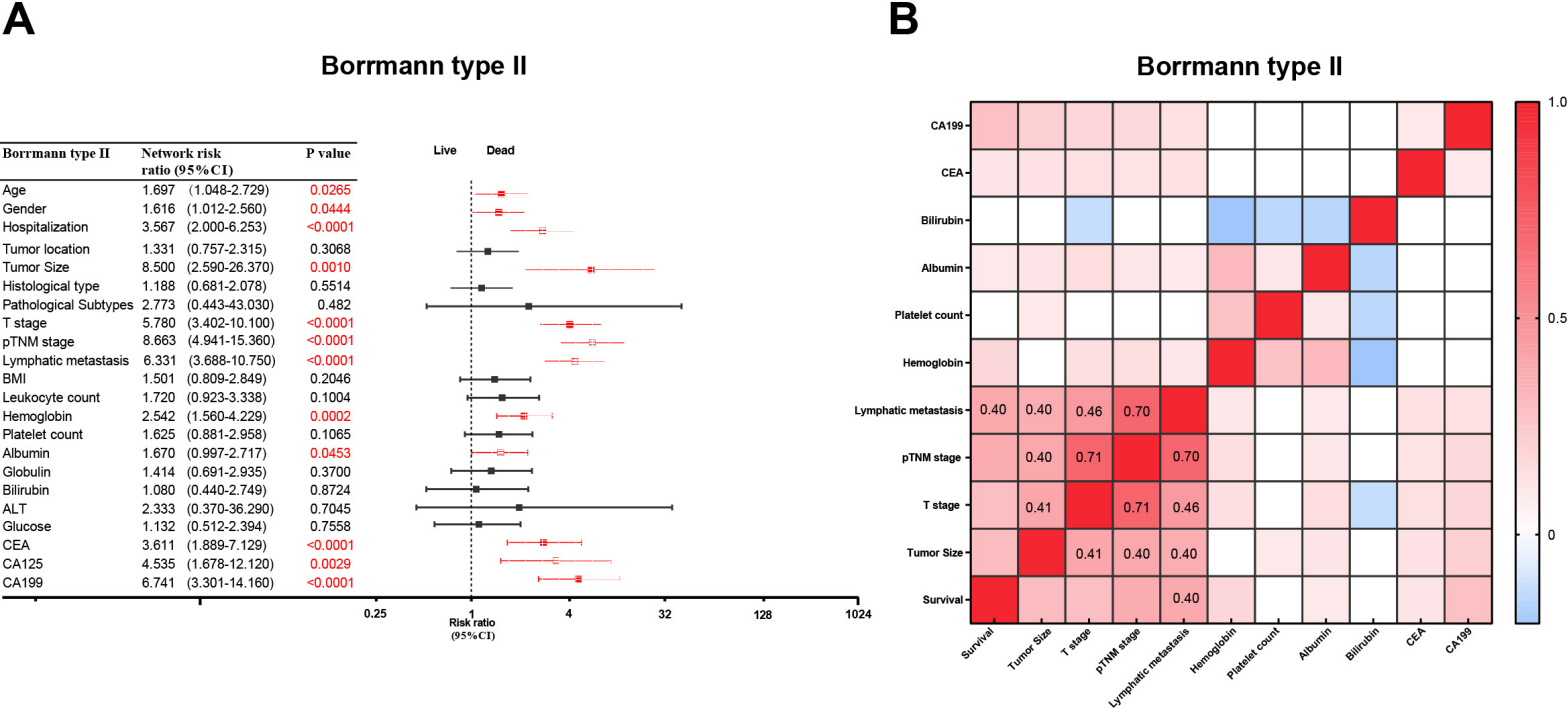
Hazard ratios and correlation networks of type II clinicopathologic characteristics. **(A)** RRs of clinicopathologic characteristics for type II GC patients. Symbols and error bars: black, P>0.05; blue, red, P<0.05. **(B)** The correlation networks show different profiles of correlations among the clinicopathologic characteristics of type II GC patients. Kendall's tau-b test was used for correlation analysis.

Patients with type II GC presented several individual risk factors influencing survival outcomes (**Figure 4A**). Notably, factors such as tumor size (hazard ratio [HR] = 8.500, 95% confidence interval [CI]: 2.590–26.370, p = 0.0010), T stage (HR = 5.780, 95% CI: 3.402–10.100, p < 0.0001), lymphatic metastasis (HR = 6.331, 95% CI: 3.688–10.750, p < 0.0001), pathological TNM (pTNM) stage (HR = 8.663, 95% CI: 4.941–15.360, p < 0.0001), and elevated CA19–9 levels (HR = 6.741, 95% CI: 3.301–14.160, p < 0.0001) were significantly associated with poorer survival outcomes (**Figure 4A**). Similar results were obtained in the validation cohort, further confirming the reliability of these associations (**Supplementary Figure 2B**). Moreover, through systematic analysis and comparison with types I, III, and IV gastric cancer, we discovered that type II gastric cancer exhibits a unique and complex spectrum of prognostic influencing factors, which strongly supports its management and investigation as a distinct clinical entity. (**Supplementary Figures 2C–E**).

Among these factors, lymphatic metastasis demonstrated a notable positive correlation with adverse survival outcomes (r = 0.40, **Figure 4B**). Furthermore, lymphatic metastasis was moderately correlated with both the T stage (r = 0.40) and the pTNM stage (r = 0.70) (**Figure 4B**). These findings suggest that lymphatic metastasis may play a pivotal role as a critical risk factor in type II GC.

### 3.4 Increased proportion of certain risk factors in the recurrent subgroup of type II GC

It is well established that tumor recurrence significantly impacts patient prognosis. In this study, patients with recurrent type II GC demonstrated markedly worse survival outcomes than their nonrecurrent counterparts did (**Figure 2C**). To further investigate the incidence of recurrence in type II GC patients, we excluded patients with stage IV disease, ultimately enrolling 422 postoperative patients for additional analysis. Among these patients, 6.9% (n = 29) developed recurrent GC during follow-up (**Table 2**).

**Table 2:** Comparison of clinicopathological characteristics between recurrent and non-recurrent patients with Borrmann type II gastric cancer.

2007-2019	Borrmann II	No recurrence		Recurrence		p value
Proportion	422	393	(93.1%)	29	(6.9%)	
Survival (months)	22.1(9.8-65.9)	40.0(18.5-71.5)		36.0(22.5-70.5)		n.s.
Survival or death rate (n)						
Live	341	332	(84.5%)	9	(31.0%)	<0.0001
death	81	61	(15.5%)	20	(69.0%)	
Age, years	62(54-70)	59(49-66)		59(54-66)		n.s.
<45	68	64	(16.3%)	4	(13.8%)	n.s.
45-59	155	143	(36.4%)	12	(41.4%)	
≥60	199	186	(47.3%)	13	(44.8%)	
Gender						n.s.
Male, n (%)	263	244	(62.1%)	19	(65.5%)	
Female, n (%)	159	149	(37.9%)	10	(34.5%)	
Hospitalization, days	10(8-13)	9(8-12)		10(8-11)		n.s.
≤8, n (%)	164	154	(39.2%)	10	(34.5%)	n.s.
9-15, n (%)	211	193	(49.1%)	18	(62.1%)	
>15, n (%)	47	46	(11.7%)	1	(3.4%)	
Tumor location, n (%)						
Upper stomach	92	80	(20.7%)	12	(44.5%)	0.0034
Corpus	80	75	(19.4%)	5	(18.5%)	
Lower stomach	242	232	(59.9%)	10	(37.0%)	
Tumor Size (cm)	4.00(3.00-6.00)	2.00(1.5-3.00)		3.00(2.00-4.00)		
Histological type						
well-differentiation	14	14	(4.7%)	0	(0.0%)	n.s.
moderately differentiation	100	94	(31.3%)	6	(24.0%)	
poor-differentiation	211	192	(64.0%)	19	(76.0%)	
T stage						
T2	229	223	(59.9%)	6	(21.4%)	
T3	152	133	(35.8%)	19	(67.9%)	
T4	19	16	(4.3%)	3	(10.7%)	<0.0001
pTNM stage						
I	146	143	(36.4%)	3	(10.3%)	
II	107	102	(26.0%)	5	(17.2%)	
III	169	148	(37.7%)	21	(72.4%)	<0.0001
Lymphatic metastasis	2(0-5)	0(0-2)		3(0-7)		
N0	247	239	(60.8%)	8	(27.6%)	
Node-positive	175	154	(39.2%)	21	(72.4%)	0.0005
BMI	0.21(0.19-0.24)	0.22(0.20-0.24)		0.22(0.19-0.24)		n.s.
<0.185 (low weight)	36	33	(11.1%)	3	(13.6%)	n.s.
0.185-0.25 (normal)	232	216	(73.0%)	16	(72.7%)	
0.25-0.3 (overweight) & >0.3 (obesity)	50	47	(15.9%)	3	(13.6%)	
Laboratory findings						
Hematologic						
Leukocyte count, 10^9^/l	6.16(5.16-7.16)	5.86(4.87-7.14)		5.28(4.68-7.16)		n.s.
Hemoglobin, g/l	115(81-137)	127(107-143)		121(97-133)		n.s.
Platelet count, 10^9^/l	232(187-344)	234(189-276)		231(170-310)		n.s.
Albumin, g/l	38.00(35.70-40.60)	40.00(37.00-43.00)		39.10(37.23-42.85)		n.s.
Globulin, g/l	25.00(22.30-28.60)	25.70(23.00-29.00)		24.65(22.58-28.03)		n.s.
Glucose, mmol/l	4.90(4.40-5.60)	4.80(4.40-5.30)		4.60(4.28-5.13)		n.s.
AFP, ug/l	2.92(2.03-4.01)	2.76(2.03-3.52)		2.96(2.09-4.10)		n.s.
CEA, ug/l	2.24(1.48-4.92)	1.93(1.27-3.16)		1.99(1.58-3.90)		n.s.
CA125, U/ml	10.25(6.85-17.93)	9.45(6.90-12.80)		9.70(6.90-12.20)		n.s.
CA19-9, U/ml	11.52(3.32-73.61)	6.33(2.98-13.73)		7.72(2.00-14.92)		n.s.
SCC, ug/l	0.70(0.60-1.23)	0.70(0.50-0.90)		0.60(0.40-0.90)		n.s.

Analysis of preoperative characteristics revealed that recurrent patients presented distinct clinicopathological features compared with nonrecurrent patients. Specifically, their tumors were more frequently located in the upper stomach (44.5% vs. 20.7%, p = 0.0034), infiltrated deeper into the gastric wall (10.7% vs. 4.3%, p < 0.0001), and presented with more advanced pathological TNM (pTNM) stages (72.4% vs. 37.7%, p < 0.0001) (**Table 2**). Additionally, recurrent patients had larger tumors and more extensive lymphatic metastasis (p = 0.0089 and p < 0.0001, respectively, **Figures 3H, I**). To account for the limited number of recurrent cases and potential small-sample bias, we applied Firth’s penalized logistic regression with 5000 bootstrap resamples. This analysis identified pathological N stage, pathological T stage, and pTNM stage as robust predictors of recurrence, whereas tumor location and tumor size showed weaker associations (**Table 3**). However, the limited number of recurrent cases precluded further prognostic stratification analysis.

**Table 3:** Firth’s penalized logistic regression analysis with bootstrap resampling for predictors of recurrence in Borrmann type II gastric cancer.

Borrmann II	OR	95% CI	Significance frequency (p<0.05)
General pathological predictors			
Pathological N stage	2.70	1.57 – 5.12	95.1%
Pathological T stage	4.85	1.30 – 15.21	74.0%
pTNM stage (I–III)	4.04	1.91 – 15.47	98.0%
Tumor location (Upper vs Corpus)	1.53	0.59 – 4.60	12.9%
Tumor size	1.26	1.06 – 1.54	50.6%
Specific lymph node station involvement			
LN_No. 1	9.46	3.85 – 19.23	99.8%
LN_No. 2	6.85	2.23 – 15.11	89.9%
LN_No. 3	7.01	2.95 – 14.65	99.5%
LN_No. 7	5.48	2.07 – 11.43	93.1%

OR, odds ratio; CI, confidence interval. Bootstrap resampling with B = 5000 iterations was applied. Firth’s penalized logistic regression was used to reduce small-sample bias.

**Figure 5:**
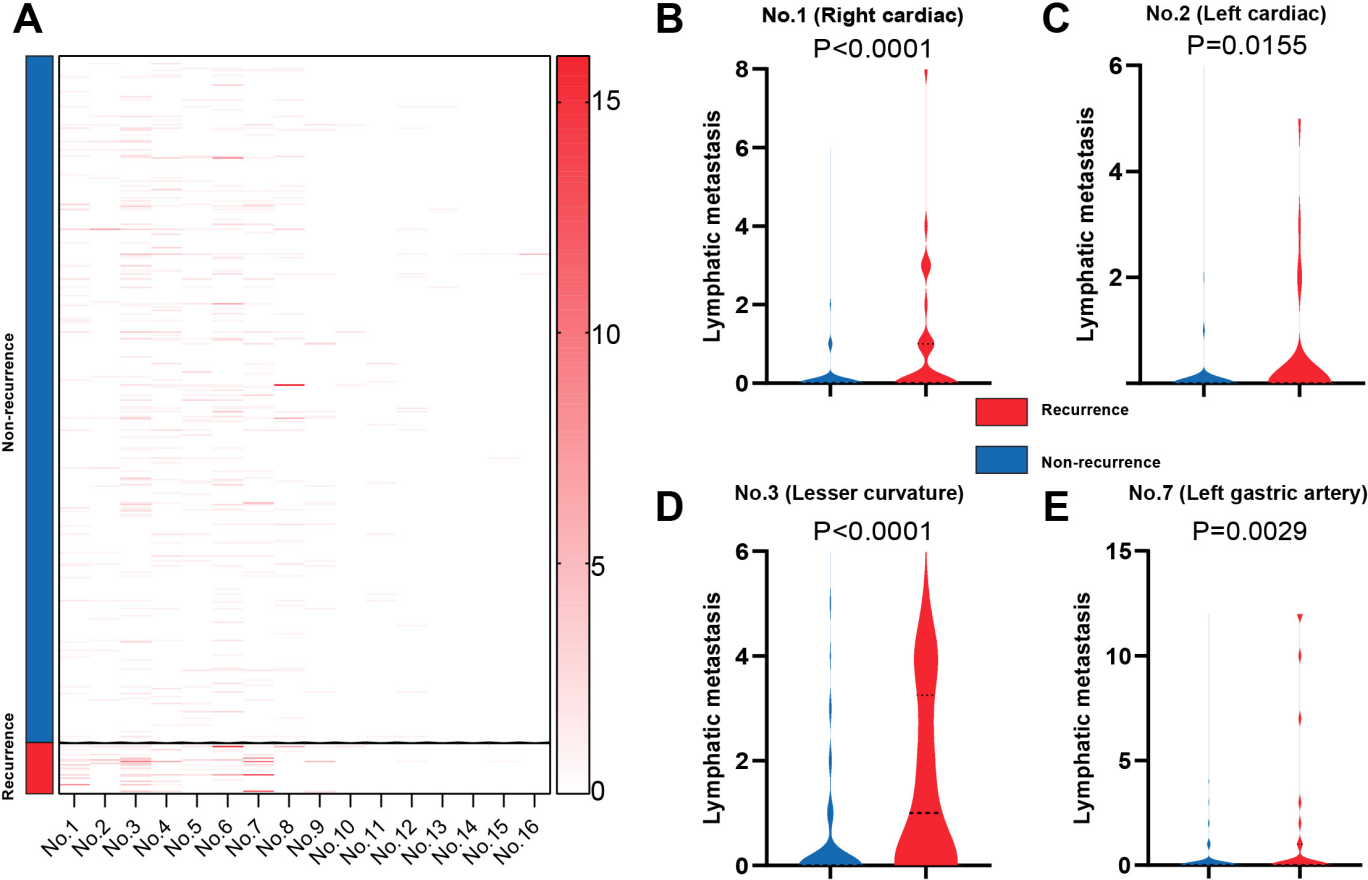
Comparison of the positive LN group between the recurrent and nonrecurrent groups. **(A)** Heatmap showing the frequencies of positive LNs from the No. 1 group to the No. 16 group. **(B–E)** Comparison of positive LNs between the recurrent and nonrecurrent groups in the No. 1 group, No. 2 group, No. 3 group, and No. 7 group.

Previous analyzes revealed that lymphatic metastasis is a critical risk factor limiting the prognosis of type II GC patients. To explore the relationship between recurrence and regional lymph node (LN) status, we found that recurrent patients presented a greater frequency of regional LN metastasis than nonrecurrent patients did (**Figure 5A**). Specifically, the first LN station (including No. 1, No. 2, and No. 3) and the second LN station (No. 7) demonstrated significantly higher rates of positive LN metastasis in the recurrent group than in the nonrecurrent group (p < 0.001, p = 0.0155, p < 0.0001, and p = 0.0029, respectively, **Figures 5B–E**). These findings were further validated by resampling analysis of LN stations 1, 2, 3, and 7, which yielded consistent results (**Table 3**). In the validation cohort, similar distributional patterns were observed, supporting the generalizability of these findings despite the absence of statistical testing (**Table 4**).

**Table 4:** Validation of specific regional lymph node involvement between recurrent and non-recurrent Borrmann type II gastric cancer patients.

LN station	Recurrent (n = 5)	Nonrecurrent (n =39)	Positive rate (%)	Direction vs. external center
No. 1	2/5	13/39	40.0 vs. 33.3	Consistent
No. 2	3/5	8/39	60.0 vs. 20.5	Consistent
No. 3	3/5	7/39	60.0 vs. 17.9	Consistent
No. 7	2/5	8/39	40.0 vs. 20.5	Consistent

Data are presented as the number of positive cases/total number (percentage). No statistical tests were performed due to the small number of recurrence events (n = 5).

## 4 Discussion

The Borrmann classification system has been widely utilized for stratifying GCs for decades, with macroscopic features identifiable via preoperative endoscopy (6, 20). This system can aid surgeons in assessing GC prognosis and determining treatment strategies. For example, while type III GC typically presents with poorly defined margins, both type I and type II tumors exhibit sharp demarcations, with type II tumors characterized as the ulcerated type (14). Owing to its grave prognosis, type IV GC has garnered the most research attention. Approximately 50% of GC cases are type IV, making it a critical independent prognostic factor (10, 21, 22). However, type IV GC is sometimes misdiagnosed as gastric lymphoma, even by experienced clinicians (8, 23). Consequently, type IV (12.8%) patients were excluded from our statistical comparison of type II patients with other Borrmann types.

Tumor size is a recognized prognostic factor for Borrmann type III GC, with outcomes significantly deteriorating when tumors exceed 8 cm (23). Conversely, type I GC has a higher recurrence rate than type II and III GC does, which is attributed to its larger tumor size (17). Despite extensive investigations into Borrmann classifications, few studies have focused on type II GC. In this study, we retrospectively analyzed the clinicopathological and prognostic characteristics of type II GC patients in a large cohort. Furthermore, we evaluated potential risk factors for recurrence among type II patients. Notably, type II patients were enrolled across all pTNM stages compared with type I and III patients, but stage IV patients (3.0%) were excluded when recurrence was analyzed.

Kim et al. reported that type II Borrmann types accounted for 30.3% of Borrmann types in a Japanese cohort, a proportion that is consistent with our findings (24.1%). Recent studies suggest that survival outcomes for type II GC patients are either inferior or comparable to those of type I patients (13, 17). However, our analyzes of both the primary and validation cohorts consistently showed that type II had the best prognosis among all Borrmann types. This apparent “survival paradox”—wherein type II tumors demonstrated superior prognosis relative to type I in our cohort, contrary to select Japanese reports—likely reflects regional disparities in endoscopic screening intensity and therapeutic algorithms. In regions with rigorous screening programs (e.g., Japan), early-stage type I lesions are frequently identified and definitively managed via endoscopic submucosal dissection (24, 25). Consequently, type I cases ultimately referred for radical gastrectomy represent a highly selected subset of advanced or biologically aggressive disease, thereby skewing surgical outcomes toward poorer survival. By contrast, the paucity of type I cases in our series (3.3%) likely mirrors this selection bias. Additionally, the sharply circumscribed margins characteristic of type II tumors may facilitate the attainment of negative resection margins more readily than infiltrative subtypes, further contributing to the observed survival advantage. Notably, type II tumors are characterized by deep ulcers, elevated edges, and localized infiltration (17), characteristics that may facilitate deep tissue invasion and lymphatic metastasis. When these tumors invade the serosal layer, peritoneal metastasis may occur, a phenomenon that aligns with our finding that peritoneal metastasis was the most common recurrence pattern (37.9%) in type II patients. Competing risk regression was applied to identify site-specific predictors, but no independent factors emerged, likely reflecting the limited number of recurrent cases. Larger, adequately powered studies are warranted to refine risk stratification and guide tailored surveillance and therapeutic strategies.

More importantly, through systematic multivariate analysis and comparisons with types I, III, and IV disease, we revealed the uniqueness of the prognosis of Borrmann type II disease. Compared with those of other subtypes, the survival outcomes of type II patients are not only significantly influenced by traditional pathological staging (such as T stage, pTNM stage, and lymphatic metastasis—factors that are universally important across all subtypes), but also uniquely governed by a combination of patient baseline characteristics (e.g., age and sex), clinical nutritional status (e.g., low BMI and low albumin), and tumor markers (e.g., CA125 and CA19-9). This multilayered pattern of influencing factors was not observed in types I, III, or IV, highlighting the complexity of the prognosis of Borrmann type II.

This study confirmed that, compared with nonrecurrent patients, recurrent type II GC patients presented with larger tumors, higher rates of lymphatic metastasis, and a greater propensity for upper stomach localization. Tumor size has been identified as a prognostic factor across several malignancies, including breast and lung cancers, although it is not explicitly considered in GC staging (26). Previous studies have demonstrated that tumor burden, reflected by tumor size, significantly impacts GC prognosis (27, 28). Wang et al. (29) identified tumor size as an independent prognostic factor, whereas Kei et al. reported its association with poor survival in type III GC and high recurrence rates in type I GC (17, 27). Our study also revealed that tumor size was correlated with T stage, lymphatic metastasis, and pTNM stage, emphasizing its role in the assessment of recurrence risk for type II GC.

Lymphatic metastasis is another critical prognostic factor for GC (30–33). In our study, compared with nonrecurrent patients, recurrent type II patients had significantly higher rates of Node-positive and upper stomach tumors and a greater prevalence of positive lymph node (LN) metastasis at stations No. 1, No. 2, No. 3, and No. 7. These outcomes may result from two key factors: (1) Upper GCs generally have worse outcomes than the corpus or lower GCs do (12), as right paracardial nodes (No. 1) and left gastric artery nodes (No. 7) facilitate early lymphatic dissemination (34, 35). (2) No. 7 LNs serve as a critical drainage pathway at the second LN station, with tumor cells progressing to more distant sites once they traverse the first station (12, 36). No. 7 LN metastasis, reported in 14.82% of GC patients, is associated with significantly poorer outcomes (37).

Interestingly, our findings highlighted the prognostic relevance of body mass index (BMI) and the serum ALB concentration. Consistent with prior studies (18, 38), low preoperative BMI and hypoalbuminemia were associated with poor long-term outcomes in type II GC patients. Malnutrition and weight loss are common in patients with GC and contribute to adverse prognoses (19, 39). The level of albumin, a nutritional marker, reflects disease progression and survival (40, 41). Abnormal ALB levels have been linked to GC and reduced overall survival (42, 43), findings that align with our observations.

Although Borrmann type II gastric cancer generally has a favorable prognosis, recurrence remains a prominent issue. In this study, two patients with T1N0M0 Borrmann type II disease experienced recurrence postoperatively, indicating that even early-stage patients are at risk of recurrence. Multicenter data and various statistical methods have shown that lymph node metastasis is a key risk factor for recurrence in type II patients. This underscores the critical importance of meticulous lymphadenectomy during surgery, especially with respect to Borrmann type II gastric cancer, where the clearance of second-tier lymph nodes should be emphasized. Among these nodes, the lymph nodes at station 7, which are distributed along the left gastric artery, may be key to recurrence. Careful intraoperative assessment and complete excision of these lymph nodes can significantly reduce recurrence rates and improve long-term survival. The guidelines for performing D2 lymphadenectomy vary across countries, and these discrepancies may contribute to differing recurrence rates in different regions (1, 25, 44, 45). Increasing the number of standardized lymphadenectomy protocols could optimize the prognosis of patients with type II gastric cancer. Patients with Borrmann type II gastric cancer accompanied by involvement of the 7th lymph node, upper gastric tumors, or malnutrition should be classified as high risk and monitored more intensively, with consideration for adjuvant therapy. Preoperative nutritional assessment and postoperative supportive care are critical for improving patient prognosis.

This study has several limitations. First, regarding the non-associations in this study, the small number of recurrent events in the validation cohort restricted our statistical power to independently validate the predictive value of No. 7 LN status. While this remains an unconfirmed association in our external series, the high degree of directional consistency across centers underscores its potential as a clinical red flag for high-risk type II patients. Second, the unavailability of data regarding postoperative complications and adjuvant chemotherapy regimens represents a notable limitation. Given that standard adjuvant chemotherapy significantly influences survival and recurrence in advanced gastric cancer, the absence of these treatment details introduces potential confounding that may affect survival analyzes. Third, key biomarker data—including HER2 status, microsatellite instability and PD-L1 expression—were unavailable. This paucity of molecular profiling is particularly consequential, as these markers likely underlie the distinct biological behaviors observed in Borrmann type II tumors. For instance, the characteristic deep local infiltration and sharply circumscribed ulceration of type II lesions may reflect specific molecular subtypes (e.g., MSI-high tumors) or distinct immune microenvironments (e.g., PD-L1–positive status). Without these data, our ability to fully elucidate the biological mechanisms driving aggressive lymphatic spread and subsequent recurrence in this macroscopically “favorable” subset is constrained. Fourth, this study included only treatment-naive patients who underwent upfront radical gastrectomy. While this ensures homogeneity in pathological assessment, our findings may not be generalizable to patients who received neoadjuvant therapy, which has become increasingly standard for locally advanced gastric cancer in recent years. Finally, the limited number of recurrent events reduced statistical power to examine recurrence patterns in relation to specific lymph node status.

## 5 Conclusions

In conclusion, while type II gastric cancer has the best prognosis among all Borrmann types, it is characterized by a unique and complex spectrum of prognostic factors. Tumors located in the upper stomach, advanced T3 stage, lymphatic metastasis, low BMI, and elevated levels of CEA and CA19–9 are associated with poorer survival, and disease recurrence dramatically impairs prognosis. Further analysis revealed that lymphatic metastasis, particularly within the No. 7 lymph node group among the second-tier lymph node stations, was a critical risk factor influencing the survival outcomes of recurrent type II patients.

## Data Availability

The original contributions presented in the study are included in the article/**Supplementary Material**. Further inquiries can be directed to the corresponding authors.

## Supplementary material

Supplementary Figure 1 K–M survival curves of various subgroups of gastric cancer patients according to Borrmann classification. **(A)** Male; **(B)** female; **(C)** age 40–59 years; **(D)** age ≥60 years; **(E)** tumor located in the lower stomach; **(F)** hospitalization <8 days; **(G)** hospitalization 9–15 days; **(H)** adenocarcinoma; **(I)** poor differentiation; **(J)** moderate differentiation; **(K)** T2 stage; **(L)** Stage II; **(M)** Stage IV; **(N)** abnormal platelet count; **(O)** abnormal hemoglobin count; **(P)** abnormal serum albumin; **(Q)** body mass index (BMI) of type II patients; **(R)** serum albumin level of type II patients.

Supplementary Figure 2 Analyses of the validation and main cohorts. **(A, B)** Validation cohort. **(A)** Kaplan–Meier overall survival curves comparing Borrmann types, confirming the favorable prognosis of type II gastric cancer patients. **(B)** Hazard ratios of clinicopathological factors associated with survival in Borrmann type II gastric cancer patients. **(C–E)** Main cohort: Hazard ratio analyzes for Borrmann types I, III, and IV, showing that the identified risk factors were not unique to type II but were also present in other subtypes.

Supplementary Table 1 Clinicopathological characteristics and survival outcomes of gastric cancer patients from the First Affiliated Hospital of Shantou University Medical College (2014--2017) according to the Borrmann classification. Owing to the limited number of Borrmann type I cases (n = 9), these patients were excluded from the statistical comparison. Subsequent analyzes were conducted to compare only Borrmann type II (n = 44) and type III (n = 123) fractures. Categorical variables were analyzed via the chi-square test. For any cell with an expected frequency less than 5, Fisher's exact test was applied to ensure statistical validity. *: Chi-square tests were used to compare Borrmann type II and Borrmann type III with different indices. n.s: not significant.
