# Supplementary figures and images for: Regional lymph node metastasis as a risk factor for recurrence in Borrmann type II gastric cancer: particular significance of the no. 7 lymph node
Source: Front Oncol. 2026 May 8;16:1797970. doi: 10.3389/fonc.2026.1797970 (PMC13193983; doi:10.3389/fonc.2026.1797970)

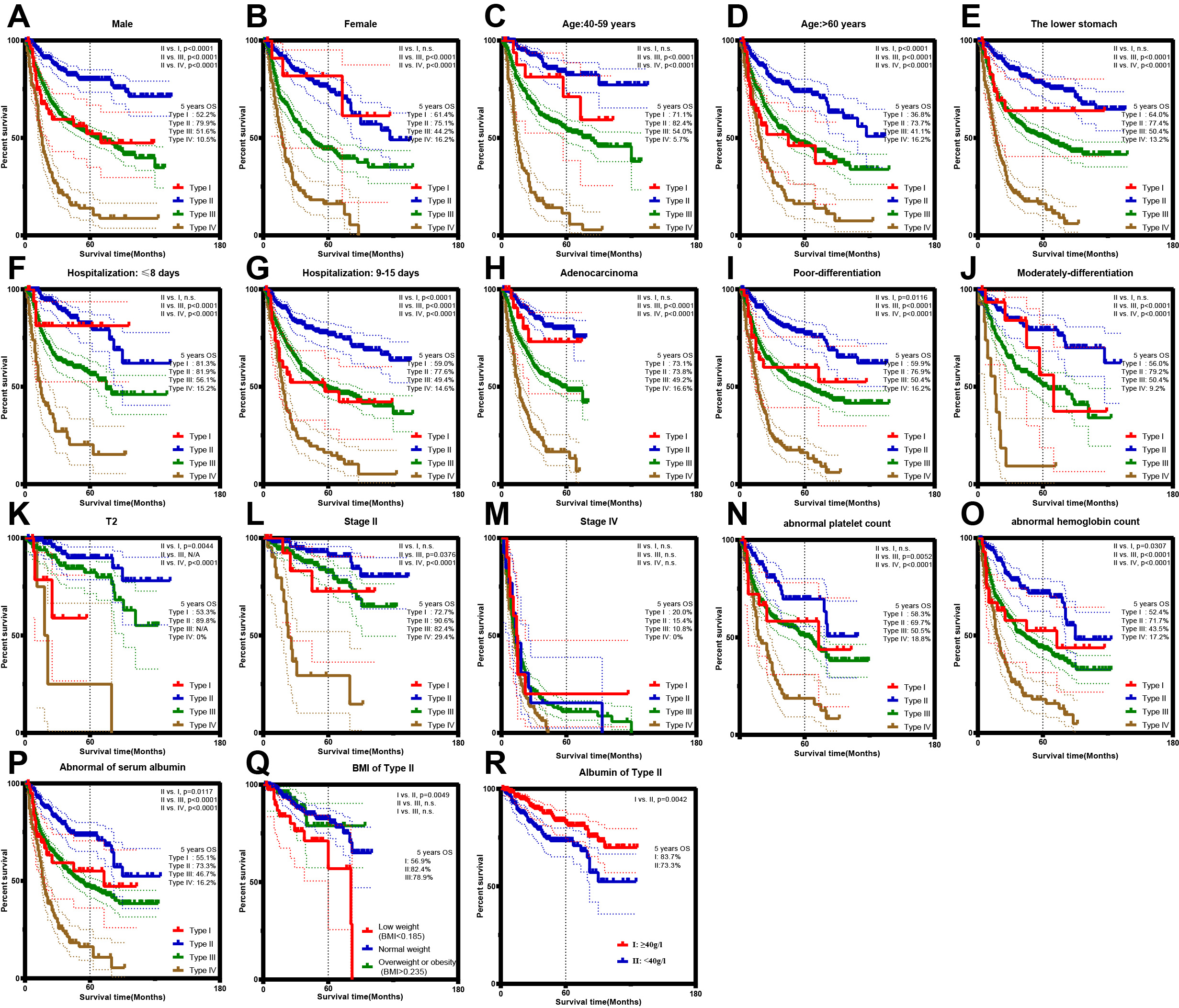

Supplement: Supplementary Figure 1 — K–M survival curves of various subgroups of gastric cancer patients according to Borrmann classification. (A) Male; (B) female; (C) age 40–59 years; (D) age ≥60 years; (E) tumor located in the lower stomach; (F) hospitalization <8 days; (G) hospitalization 9–15 days; (H) adenocarcinoma; (I) poor differentiation; (J) moderate differentiation; (K) T2 stage; (L) Stage II; (M) Stage IV; (N) abnormal platelet count; (O) abnormal hemoglobin count; (P) abnormal serum albumin; (Q) body mass index (BMI) of type II patients; (R) serum albumin level of type II patients. [file Image1.jpeg]

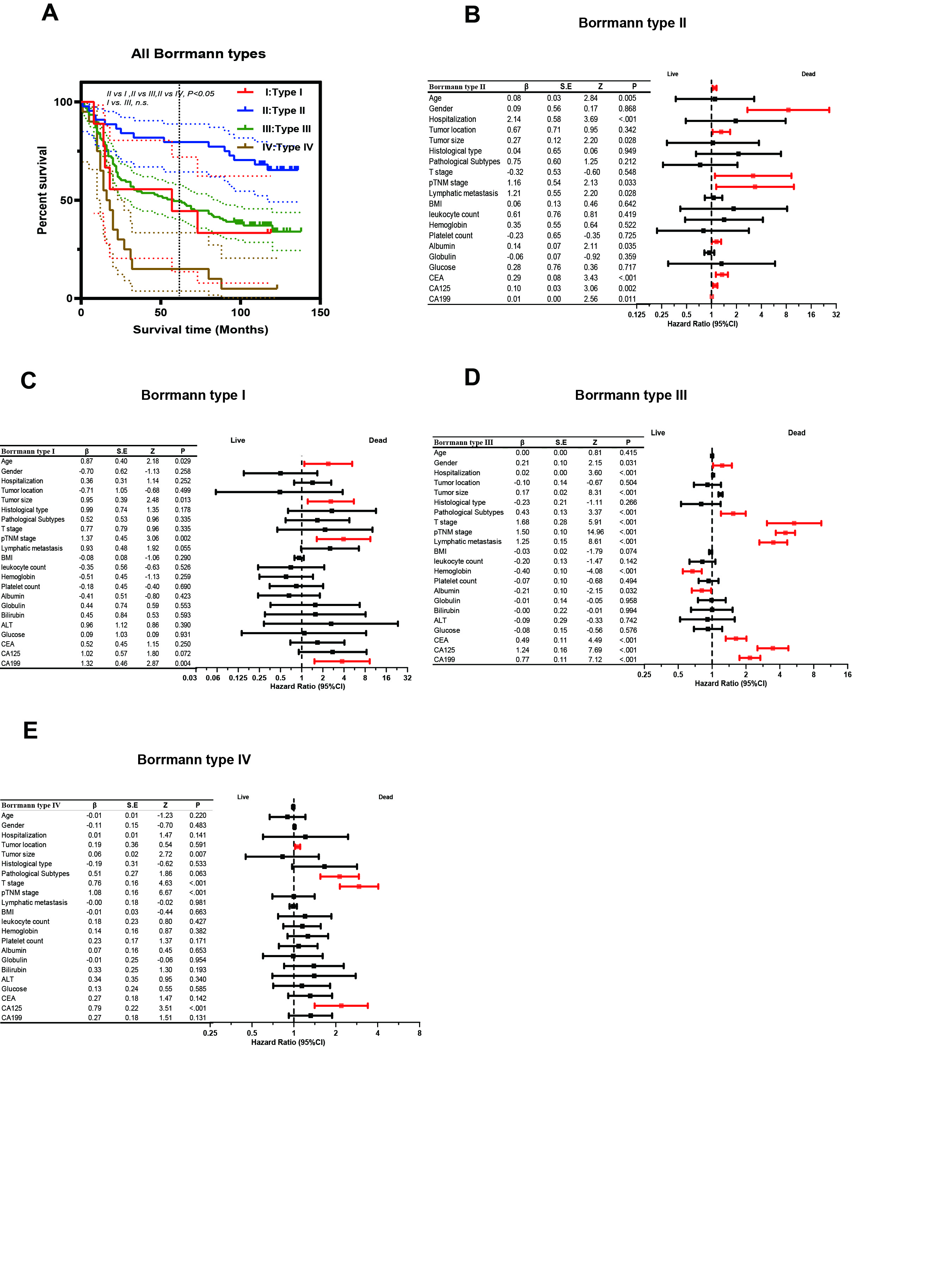

Supplement: Supplementary Figure 2 — Analyses of the validation and main cohorts. (A, B) Validation cohort. (A) Kaplan–Meier overall survival curves comparing Borrmann types, confirming the favorable prognosis of type II gastric cancer patients. (B) Hazard ratios of clinicopathological factors associated with survival in Borrmann type II gastric cancer patients. (C–E) Main cohort: Hazard ratio analyzes for Borrmann types I, III, and IV, showing that the identified risk factors were not unique to type II but were also present in other subtypes. [file Image2.jpeg]
