# Supplementary material for: Regional lymph node metastasis as a risk factor for recurrence in Borrmann type II gastric cancer: particular significance of the no. 7 lymph node
Source: Front Oncol. 2026 May 8;16:1797970. doi: 10.3389/fonc.2026.1797970 (PMC13193983; doi:10.3389/fonc.2026.1797970)
Supplement: Supplementary Table 1 — Clinicopathological characteristics and survival outcomes of gastric cancer patients from the First Affiliated Hospital of Shantou University Medical College (2014--2017) according to the Borrmann classification. Owing to the limited number of Borrmann type I cases (n = 9), these patients were excluded from the statistical comparison. Subsequent analyzes were conducted to compare only Borrmann type II (n = 44) and type III (n = 123) fractures. Categorical variables were analyzed via the chi-square test. For any cell with an expected frequency less than 5, Fisher's exact test was applied to ensure statistical validity. *: Chi-square tests were used to compare Borrmann type II and Borrmann type III with different indices. n.s: not significant. [file Table1.docx]

Supplementary Table 1: Clinicopathological characteristics and survival outcomes of gastric cancer patients from the First Affiliated Hospital of Shantou University Medical College (2014--2017) according to the Borrmann classification

| **Characteristics** | **Total** | **Borrmann type I** | | | **Borrmann type II** | | **Borrmann type III** | | **Borrmann type IV** | | **p value** |  |
| --- | --- | --- | --- | --- | --- | --- | --- | --- | --- | --- | --- | --- |
| **Proportion** | 197 | 9 | | （5.1%） | 44 | （22.3%） | 123 | （62.4%） | 20 | （10.2%） |  |  |
| **Survival or death rate（n）** | |  | |  |  |  |  |  |  |  |  |  |
| Live | 78 | 3 | | （33.3%） | 29 | （65.9%） | 45 | （36.5%） | 1 | （5.0%） |  |  |
| death | 118 | 6 | | （66.6%） | 15 | （34.0%） | 78 | （63.5%） | 19 | （95.0%） | <0.001* |  |
| **Age, years** |  |  |  |  |  |  |  |  |  |  |  |  |
| <45 | 15 | 2 | | （22.2%） | 7 | （15.9%） | 3 | （1.6%） | 2 | （10.0%） | 0.046* |  |
| 45-59 | 50 | 0 | | （0%） | 13 | （30.0%） | 29 | （23.6%） | 8 | （40.0%） |  |  |
| ≥60 | 132 | 7 | | （77.8%） | 24 | （54.5%） | 91 | （74.0%） | 10 | （50.0%） |  |  |
| **Gender** |  |  | |  |  |  |  |  |  |  |  |  |
| Male, n (%) | 152 | 4 | | （44.4%） | 29 | （66.0%） | 96 | （78.0%） | 15 | （75.0%） | n.s.* |  |
| Female, n (%) | 88 | 5 | | （55.6%） | 15 | （34.0%） | 27 | （22.0%） | 5 | （25.0%） |  |  |
| **Hospitalization, days** |  |  |  |  |  |  |  |  |  |  |  |  |
| ≤8, n (%) | 6 | 0 | | （0%） | 2 | （4.5%） | 2 | （1.6%） | 2 | （10.0%） | n.s.* |  |
| 9-15, n (%) | 133 | 8 | | （88.9%） | 31 | （70.5%） | 84 | （68.3%） | 10 | （50.0%） |  |  |
| >15, n (%) | 56 | 1 | | （11.1%） | 11 | （25.0%） | 37 | （30.1%） | 8 | （40.0%） |  |  |
| **Peritoneal metastasis, n (%)** | |  | |  |  |  |  |  |  |  |  |  |
| P0 | 186 | 8 | | （88.9%） | 42 | （95.5%） | 121 | （97.6%） | 15 | （75.0%） | n.s.* |  |
| P1 | 11 | 1 | | （11.1%） | 2 | （4.5%） | 3 | （2.4%） | 5 | （25.0%） |  |  |
| **Hepatic metastasis, n (%)** | |  | |  |  |  |  |  |  |  |  |  |
| H0 | 191 | 9 | | （100%） | 43 | （97.7%） | 121 | （98.4%） | 18 | （90.0%） | n.s.* |  |
| H1 | 9 | 0 | | （0%） | 1 | （1%） | 2 | （1.6%） | 2 | （10.0%） |  |  |
| **Tumor location, n (%)** |  |  | |  |  |  |  |  |  |  |  |  |
| Upper stomach | 82 | 2 | | （22.2%） | 14 | （31.8%） | 61 | （50.8%） | 5 | （23.8%） | n.s.* |  |
| Corpus | 49 | 3 | | （33.3%） | 14 | （31.8%） | 25 | （20.8%） | 7 | （33.3%） |  |  |
| Lower stomach | 69 | 4 | | （44.4%） | 16 | （36.4%） | 34 | （28.3%） | 9 | （42.9%） |  |  |
| **Tumor Size, n (%)** | |  | |  |  |  |  |  |  |  |  |  |
| <3 | 53 | 2 | | （25.0%） | 10 | （23.8%） | 37 | （30.8%） | 4 | （23.5%） | 0.0073* |  |
| 3-6 | 90 | 3 | | （37.5%） | 27 | （64.3%） | 56 | （46.7%） | 4 | （23.5%） |  |  |
| ≥6 | 44 | 3 | | （37.5%） | 5 | （11.9%） | 27 | （22.5%） | 9 | （52.9%） |  |  |
| **Histological type** |  |  | |  |  |  |  |  |  |  |  |  |
| well-differentiation | 7 | 1 | | （11.1%） | 2 | （4.7%） | 4 | （4.2） | 0 | （0.5%） | 0.028* |  |
| moderately differentiation | 71 | 6 | | （66.7%） | 16 | （37.2%） | 48 | （50.0%） | 1 | （7.7%） |  |  |
| poor-differentiation | 82 | 2 | | （22.2%） | 25 | （58.1%） | 44 | （45.8%） | 12 | （92.3%） |  |  |
| **Pathological Subtypes** |  |  | |  |  |  |  |  |  |  |  |  |
| Adenocarcinoma | 147 | 5 | | （55.6%） | 40 | （90.9%） | 92 | （83.6%） | 10 | （66.7%） | n.s.* |  |
| Others | 31 | 4 | | （44.4%） | 4 | （9.1%） | 18 | （16.4%） | 5 | （33.3%） |  |  |
| **T stage** |  |  | |  |  |  |  |  |  |  |  |  |
| T2 | 10 | 1 | | （11.1%） | 4 | （11.4%） | 5 | （4.6%） | 0 | （0%） | 0.024* |  |
| T3 | 75 | 6 | | （66.7%） | 5 | （14.3%） | 61 | （56.0%） | 3 | （42.9%） |  |  |
| T4 | 75 | 2 | | （22.2%） | 26 | （74.3%） | 43 | （39.4%） | 4 | （57.1%） |  |  |
| **pTNM stage** |  |  | |  |  |  |  |  |  |  |  |  |
| I | 14 | 1 | | （8.3%） | 5 | （12.5%） | 8 | （6.9%） | 0 | （0%） | 0.025* |  |
| II | 25 | 1 | | （8.3%） | 12 | （30.0%） | 12 | （10.3%） | 0 | （0%） |  |  |
| III | 104 | 0 | | （0%） | 18 | （45.0%） | 78 | （67.2%） | 8 | （50.0%） |  |  |
| IV | 41 | 10 | | （83.3%） | 5 | （12.5%） | 18 | （15.5%） | 8 | （50.0%） |  |  |
| **Lymphatic metastasis** |  |  |  |  |  |  |  |  |  |  |  |  |
| N0 | 53 | 2 | | （22.2%） | 23 | （54.8%） | 22 | （18.6%） | 6 | （33.3%） | 0.0014* |  |
| Node-positive | 145 | 7 | | （77.8%） | 19 | （45.2%） | 96 | （81.4%） | 12 | （66.7%） |  |  |
| **Laboratory findings** |  |  | |  |  |  |  |  |  |  |  |  |
| **Hematologic** |  |  | |  |  |  |  |  |  |  |  |  |
| **Leukocyte count, 10^9^/l** |  |  |  |  |  |  |  |  |  |  |  |  |
| <4 × 10^9^/l, n (%) | 11 | 0 | | （0%） | 2 | （4.5%） | 5 | （4.1%） | 4 | （20.0%） | n.s.* |  |
| 4–10 × 10^9^/l, n (%) | 156 | 8 | | （88.9%） | 35 | （79.5%） | 100 | （81.3%） | 13 | （65.0%） |  |  |
| >10 × 10^9^/l, n (%) | 29 | 1 | | （11.1%） | 7 | （15.9%） | 18 | （14.6%） | 3 | （15.0%） |  |  |
| **Hemoglobin, g/l** |  |  |  |  |  |  |  |  |  |  |  |  |
| ＞120 | 99 | 1 | | （49.1%） | 25 | （56.8%） | 64 | （52.0%） | 9 | （45.0%） | n.s.* |  |
| 90-120 | 60 | 3 | | （19.3%） | 13 | （29.5%） | 37 | （30.0%） | 7 | （35.0%） |  |  |
| <90 | 37 | 5 | | （31.6%） | 6 | （13.6%） | 22 | （17.9%） | 4 | （20.0%） |  |  |
| **Platelet count, 10^9^/l** |  |  |  |  |  |  |  |  |  |  |  |  |
| <100 × 109/l, n (%) | 1 | 0 | | （0%） | 1 | （2.3%） | 0 | （0%） | 0 | （0%） | n.s.* |  |
| 100–300 × 109/l, n (%) | 143 | 3 | | （33.3%） | 36 | （81.8%） | 90 | （73.8%） | 14 | （70.0%） |  |  |
| >300 × 109/l, n (%) | 51 | 6 | | （66.7%） | 7 | （15.9%） | 32 | （26.2%） | 6 | （30.0%） |  |  |
| **Albumin, g/l** |  | |  |  |  |  |  |  |  |  |  |  |
| <40 | 147 | 2 | | （22.2%） | 35 | （79.5%） | 94 | （76.4%） | 16 | （80.0%） | n.s.* |  |
| ≥40 | 49 | 7 | | （77.8%） | 9 | （20.5%） | 29 | （23.6%） | 4 | （20..0%） |  |  |
| **Globulin, g/l** |  | |  |  |  |  |  |  |  |  |  |  |
| ≤20 | 5 | 1 | | （11.1%） | 0 | （0%） | 4 | （3.3%） | 0 | （0%） | 0.038* |  |
| ＞20 | 191 | 8 | | （88.9%） | 44 | （100%） | 119 | （96.7%） | 20 | （100%） |  |  |
| **Glucose, mmol/l** |  |  |  |  |  |  |  |  |  |  |  |  |
| ≤6.1 | 156 | 7 | | （77.8%） | 37 | （84.1%） | 98 | （79.7%） | 14 | （70.0%） | n.s.* |  |
| ＞6.1 | 40 | 2 | | （22.2%） | 7 | （15.9%） | 25 | （20.3%） | 6 | （30.0%） |  |  |
| **AFP, ug/l** |  |  |  |  |  |  |  |  |  |  |  |  |
| ≤25 | 178 | 9 | | （100.0%） | 41 | （97.6%） | 109 | （98.2%） | 19 | （95.0%） | n.s.* |  |
| ＞25 | 11 | 0 | | （0.0%） | 1 | （2.4%） | 2 | （1.8%） | 1 | （5.0%） |  |  |
| **CEA, ug/l** |  |  |  |  |  |  |  |  |  |  |  |  |
| ≤25 | 172 | 9 | | （100%） | 41 | （97.6%） | 105 | （92.9%） | 17 | （85.0%） | n.s.* |  |
| ＞25 | 40 | 0 | | （0%） | 1 | （2.4%） | 8 | （7.1%） | 3 | （15.0%） |  |  |
| **CA125, U/ml** |  | |  |  |  |  |  |  |  |  |  |  |
| ≤35 | 106 | 9 | | （100%） | 40 | （95.2%） | 39 | （92.9%） | 18 | （90.0%） | n.s.* |  |
| ＞35 | 15 | 0 | | （0%） | 2 | （4.8%） | 3 | （7.1%） | 2 | （10.0%） |  |  |
| **CA19-9, U/ml** |  |  |  |  |  |  |  |  |  |  |  |  |
| ＜37 | 141 | 7 | | （77.8%） | 38 | （86.4%） | 84 | （82.4%） | 12 | （75.0%） | n.s.* |  |
| ≥37 | 30 | 2 | | （22.2%） | 6 | （13.6%） | 18 | （17.6%） | 4 | （25.0%） |  |  |
| **BMI** |  |  |  |  |  |  |  |  |  |  |  |  |
| <18.5 (low weight) | 19 | 1 | | （11.1%） | 4 | （16.0%） | 12 | （18.2%） | 2 | （10%） | n.s.* |  |
| 18.5-25 (normal) | 90 | 8 | | （88.9%） | 18 | （72.0%） | 46 | （69.7%） | 18 | （90%） |  |  |
| 25-30(overweight)  & >30 (obesity) | 11 | 0 | | （0%） | 3 | （12.0%） | 8 | （12.1%） | 0 | （0%） |  |  |

Owing to the limited number of Borrmann type I cases (n = 9), these patients were excluded from the statistical comparison. Subsequent analyses were conducted to compare only Borrmann type II (n = 44) and type III (n = 123) fractures. Categorical variables were analyzed via the chi-square test. For any cell with an expected frequency less than 5, Fisher's exact test was applied to ensure statistical validity.

*: Chi-square tests were used to compare Borrmann type II and Borrmann type III with different indices.

n.s: not significant.
